# Wireless Monitoring of Induction Machine Rotor Physical Variables

**DOI:** 10.3390/s17112660

**Published:** 2017-11-18

**Authors:** Jefferson Doolan Fernandes, Francisco Elvis Carvalho Souza, Glauco George Cipriano Maniçoba, Andrés Ortiz Salazar, José Alvaro de Paiva

**Affiliations:** 1 Programa de Pós-Graduação em Engenharia Elétrica, Universidade Federal do Rio Grande do Norte, Campus Universitário Lagoa Nova, Centro de Tecnologia, CEP 59078-970 Natal, Brazil; elvis.carvalho@ifrn.edu.br (F.E.C.S.); andres@dca.ufrn.br (A.O.S.); 2 Instituto Federal de Educação, Ciência e Tecnologia do Rio Grande do Norte, Campus Parnamirim, CEP 59143-455 Parnamirim, Brazil; jefferson.fernandes@ifrn.edu.br (J.D.F.); alvaro.paiva@ifrn.edu.br (J.A.P.)

**Keywords:** wireless communication, energy harvesting, electrical machines maintenance, induction motor control systems, rotor temperature

## Abstract

With the widespread use of electric machines, there is a growing need to extract information from the machines to improve their control systems and maintenance management. The present work shows the development of an embedded system to perform the monitoring of the rotor physical variables of a squirrel cage induction motor. The system is comprised of: a circuit to acquire desirable rotor variable(s) and value(s) that send it to the computer; a rectifier and power storage circuit that converts an alternating current in a continuous current but also stores energy for a certain amount of time to wait for the motor’s shutdown; and a magnetic generator that harvests energy from the rotating field to power the circuits mentioned above. The embedded system is set on the rotor of a 5 HP squirrel cage induction motor, making it difficult to power the system because it is rotating. This problem can be solved with the construction of a magnetic generator device to avoid the need of using batteries or collector rings and will send data to the computer using a wireless NRF24L01 module. For the proposed system, initial validation tests were made using a temperature sensor (DS18b20), as this variable is known as the most important when identifying the need for maintenance and control systems. Few tests have shown promising results that, with further improvements, can prove the feasibility of using sensors in the rotor.

## 1. Introduction

Electric machines play an extremely important role in industry, many of them making up 100% of their driving power. Misuse, failure to manage maintenance, and late detection of defects can cause months of repair and result in actual production losses or overspending with replacements [1].

By monitoring the physical variables of electrical machines it is possible to perform a more efficient and more reliable maintenance. This requires the use of suitable apparatus capable of recording various phenomena, such as: vibrations; temperature; performance; acceleration; among others. Based on the knowledge and analysis of the phenomena, it is possible to indicate, in advance, any defects or failures in the machines and equipment [2,3].

It is relatively easy to measure variables in the stationary part of the motor (stator), the difficulty appears when you want to observe the phenomena that occurs in the rotor. For this, one can make use of estimations from the variables of the stator, or use sensors in the rotor which brings great challenges due to the rotation [4,5,6].

The monitoring of the physical variables of the rotor is very important for the detection of failures, as well as in the application of control systems. A widely used method for controlling induction motors (called control by rotor flow orientation) requires knowledge of the rotor time constant that is very sensitive to rotor temperature by measuring the temperature it is possible to more accurately estimate the time constant and thus improve the performance of the controller. But because of the complexity of implementation and the high cost of placing sensors in the rotor, parameter estimation methods are most commonly used to obtain values close to the exact local temperature [6].

There are basically two challenges for placing sensors in the rotor, energizing the transducer/sensor and transmitting data. With the rotation, the options found to solve these problems are:

- Wireless energizing:
  ○ Batteries [5,7,8];
  ○ Collector rings;
  ○ RFID (Radio Frequency Identification) [9];
  ○ SAW (Surface Acoustic Wave) [10];
  ○ WPT (Wireless Power Transfer) [4].
- Wireless data transmission:
  ○ RF (Radio Frequency);
    ▪ 2.4 GHz [5,7,10,11];
    ▪ 433 MHz [12].
  ○ RFID (Radio Frequency Identification) [9];
  ○ SAW (Surface Acoustic Wave) [10];
  ○ IrDA (Infrared Data Association) [8].

Energy harvesting (EH) technology could play a significant role harvesting various sources of ambient energy such as vibration, electromagnetic, thermal, etc. EH can be used to power the network nodes while storing the surplus in storage units (e.g., batteries, supercapacitor) [13,14,15,16,17].

The objective of this work is to develop a wireless embedded system for monitoring the physical variables of the rotor of electric machines. For this, the works were divided in two parts:

1. Design and develop a circuit for reading rotor variables and wireless information transmission. Following with the magnetic field effect study of the motor and analysis of packet losses in the wireless transmission.
2. Design and develop a device responsible for converting magnetic energy of the rotating field into electrical energy, which will power the circuit of the embedded system. The device consists of a magnetic generator responsible for capturing magnetic energy, an AC/DC rectifier, a regulation stage, and an energy storage module using supercapacitor.

## 2. Proposed System Overview

As previously mentioned, the objective of this work is to develop an embedded system to monitor physical variables in an induction motor rotor.

The embedded system can be characterized as combining the use of hardware and software in a device with a predefined purpose. The embedded system has the purpose of controlling processes or acting on a problem. This is done using the peripherals, which are chosen and sized based on the problem [18,19,20].

**Figure 1:**
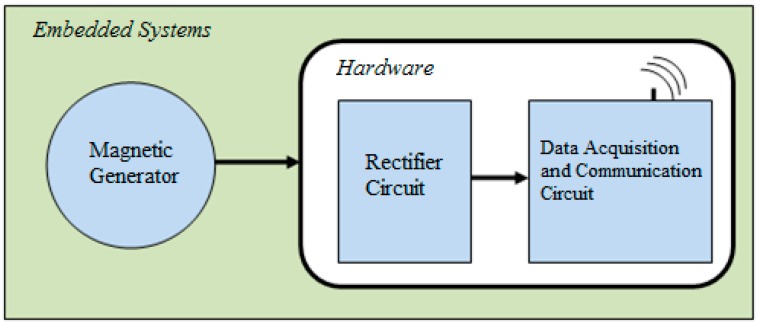
Embedded System Block Diagram.

The proposed system represented by the block diagram in Figure 1 is formed by an energy generating device and a hardware. The hardware is divided into two printed circuit boards, one to house the data acquisition and communication circuit and one to the power circuit.

### 2.1. Data Acquisition and Communication Circuit

**Figure 2:**
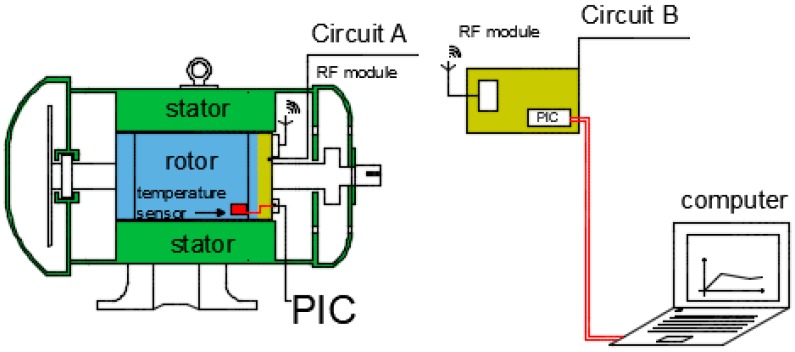
Data acquisition and communication circuit.

The data acquisition and communication circuit consists basically of transducer, microcontroller, and RF module. In this work, the structure schematically shown in Figure 2 is used, it is composed of two parts. The first is a circuit A that is coupled to the rotor and rotates along with it, here a microcontroller receives the temperature values supplied by the sensor, processes the information and sends to the RF module (Radio Frequency), it transmits the temperature values through radio frequency signals. The second part is a receiving circuit B external to the motor in which a second RF module receives the signals containing the temperature information and sends it to a microcontroller which in turn passes the information to a computer.

The characteristics of the main components will be described below where, at this point in the research, the most important information is the maximum current consumption of all components, to certify that the generating device will be able to power the circuit in consumption peak. However, component choices were made with expectation for a low power circuit.

#### 2.1.1. RF Module

**Figure 3:**
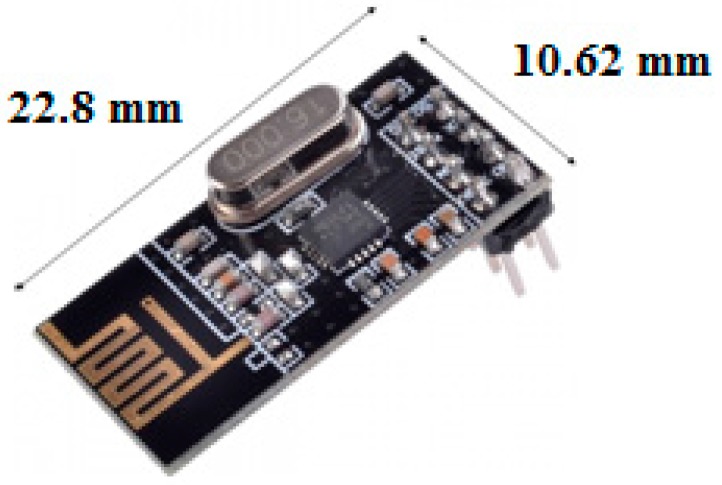
RF module NRF24L01.

To choose which RF module would be most appropriate for our application, the following criteria were considered: small size, low power consumption, low cost and the possibility to send and receive information (transceiver). The one that most approached these characteristics was the NRF24L01 of the manufacturer Nordic Semiconductor^®^, shown in Figure 3.

The NRF24L01 is a ULP (Ultra Low Power) transceiver that has a data transfer rate of 2 Mbps and operates in the 2.4 GHz frequency band. Its peak consumption in the transmission or reception of data is 14 mA.

#### 2.1.2. Microcontroller

**Figure 4:**
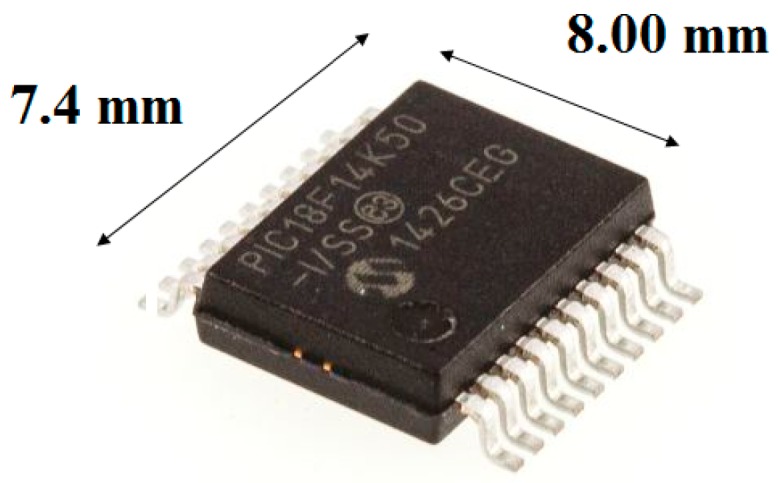
Microcontroller.

Like the RF module, the choice of the microcontroller was subject to the same criteria: reduced size; low power consumption; and low cost. It must also be compatible with the SPI communication protocol in order to exchange information with the RF module. Thus was chosen the microcontroller PIC18f14K50 from the manufacturer Microchip Technology Inc., shown in Figure 4.

A 12 MHz crystal was used as oscillator, multiplied by four in the PIC giving a processing frequency of 48 MHz. With this frequency and power of 3.3 V its estimated maximum consumption is 12 mA.

#### 2.1.3. Transducer

Several types of sensors and/or transducers can connect to the proposed embedded system, since the microcontroller used allows analog and digital inputs and supports various communication protocols (SPI, I2C, UART, 1-WIRE).

For this work the digital thermometer DS18b20, manufactured by Maxim^®^ (San José, CA, USA), was used. It is a digital transducer that provides data calibrated in degrees Celsius. It uses 1-Wire protocol communication which allows to connect several sensors in the same bus occupying a single pin of the microcontroller that allows monitoring temperature of several points of the rotor.

### 2.2. Power Circuit

To design the supply circuit and the generating device it is necessary to know the current demand of the load, that is, the acquisition and communication board. From information of the components of this, a maximum expected consumption of approximately 33 mA was reached, as shown in Table 1.

With this, it was decided to design a power circuit with output voltage of 3.3 V and capacity for a maximum current of 50 mA, leaving a margin to avoid overload.

### 2.3. Generator Device

The motor on which the embedded system was installed is a three-phase four-pole squirrel-cage rotor whose nominal parameters are listed in Table 2. From the characteristics of this motor it was dimensioned the generator device of the embedded system.

#### 2.3.1. Math Analysis

In order to make a qualitative analysis about the parameters and how they influence the induced voltage, a mathematical equation was developed based on the basic concepts of electromagnetism.

**Figure 5:**
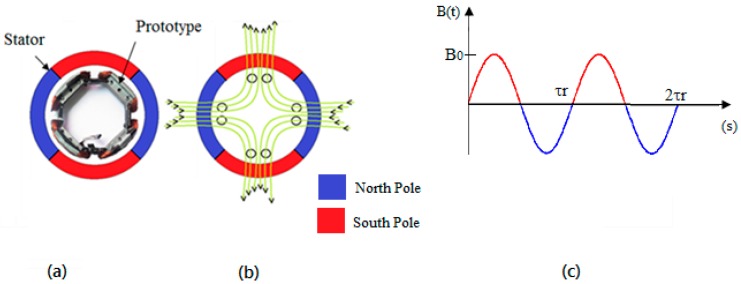
Arrangement of the generator coils in relation to the four poles generated by the stator. (**a**) Physical device; (**b**) simplified representation; (**c**) stator magnetic flux wave form.

Figure 5a,b illustrate the four-pole magnetic field within the motor at a given time point, and how the coils of the generator device interact with this magnetic field.

**Figure 6:**
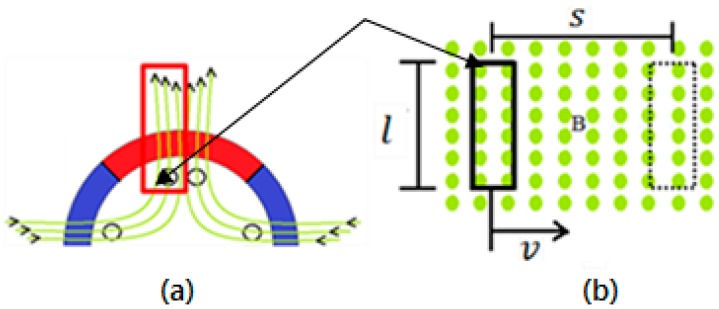
Representation of the relative displacement between coil conductors and the stator magnetic field. (**a**) Frontal view; (**b**) top view.

The rotational field traverses the coils of the generator with velocity *v*, which in a time interval ∆t travels the space *S*. Figure 6a shows a top view of Figure 6b, the small circles in b are the tips of the magnetic flux arrows in a.

Faraday’s Law states that in a conductor subjected to a magnetic flux variation *φ* an induced electromotive force arises *e_ind_* where absolute value for *N* conductors is given as:

(1) $$e_{ind}=N\frac{d\phi }{dt}.$$

Considering that the magnetic flux is perpendicular to the area swept by the conductor, it can be calculated by:

(2) ϕ=∫BdA.

The flux density *B* in the air gap, shown in Figure 5c, can be described approximately by a sinusoidal

(3) $$B(s)=B_{0}sin\left(\frac{2}{r}s\right)$$

where *B*_0_ is the maximum value assumed by *B*(*s*), *r* is the radius of the generator, and *s* is the space traveled along the circumference.

The area can be calculated by:

(4) A(s)=l.s => dA=lds.

Since *l* is the length of the conductor cutting the flux lines, see Figure 6b.

Substituting Equations (3) and (4) into (2), determine the magnetic flux:

(5) $$\phi (s)=B_{0}l\int_{0}^{s}sin\left(\frac{2}{r}s\right)ds=\frac{B_{0}lr}{2}\left[1-\cos\left(\frac{2}{r}s\right)\right].$$

Knowing that,

(6) $$s=v.t,\;\mathrm{and}\;v=\frac{\omega }{2}r.$$

In which *v* and *ω* are the linear and angular velocities of the conductor relative to the magnetic flux lines. If the rotor is rotating ω will be in function of the slip frequency. The value 2 dividing *ω* refers to the number of pole pairs.

The expression of the flux becomes:

(7) $$\phi (t)=\frac{B_{0}lr}{2}\left[1-\cos\left(\omega t\right)\right].$$

Substituting (7) into (1) and solving the derivative, we have:

(8) $$e_{ind}=\frac{NB_{0}lr\omega }{2}sin(\omega t).$$

This equation calculates the induced voltage in the conductors of one side of the coil, the induced voltage in the complete coil is twice this value, therefore:

(9) $$e_{ind}=NB_{0}lr\omega sin(\omega t).$$

This expression reveals that the maximum value of the induced voltage depends directly on the dimensions of the generator *l* and *r*, the number of turns that is limited by the dimensions, the slip frequency *ω* and *B*_0_ which is associated with the motor supply current.

#### 2.3.2. Prototype Construction

**Figure 7:**
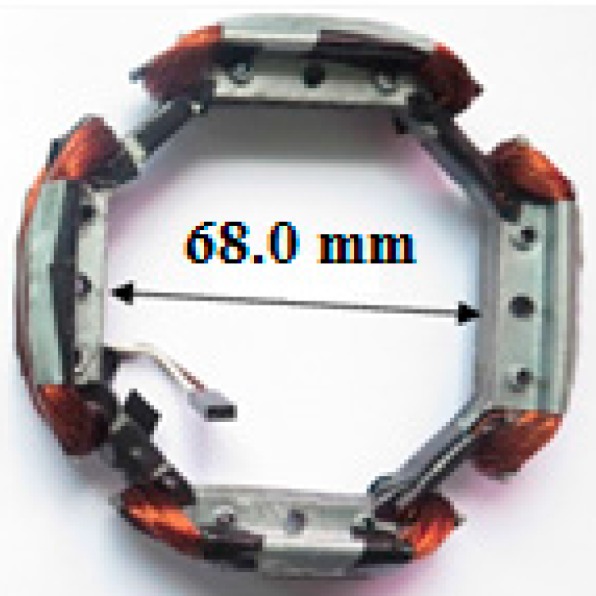
Prototype generator set.

The rotor has a diameter of 99.0 mm, so a core diameter of 96.0 mm was defined for the generator in order to avoid collisions with the stator. The generator device core was laminated to minimize eddy current losses [11,21,22]. The embedded system will be fixed to the rotor, more specifically to the extensions of the aluminum bars shown in Figure 7.

The blades were made with galvanized steel sheet number 22, material chosen for being ferromagnetic and easily accessible. Each blade is 0.80 mm thick. Twelve blades were made which, after being ready, received a layer of varnish to electrically isolate one blade from the other. The thickness of the core was approximately 10.0 mm, so as not to compromise the total available space that is 30.0 mm, because there will be 20.0 mm remaining for the coils, 10.0 mm for each side of the core.

Using 30 AWG enameled wire, four coils with 180 turns each were connected in series. Figure 7 shows the prototype ready.

#### 2.3.3. PCBs Prototyping

**Figure 8:**
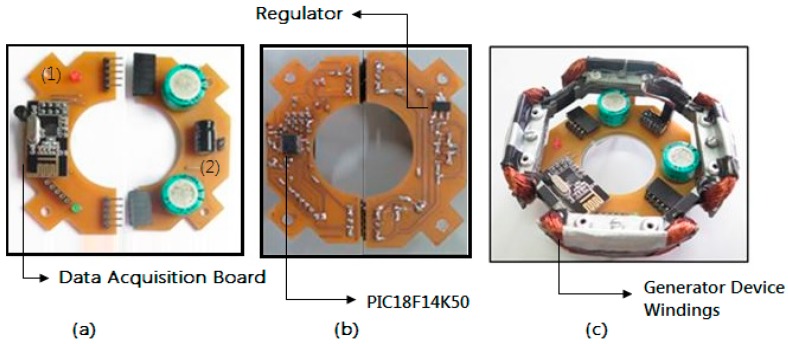
PCBs. (**a**) Top view; (**b**) bottom view; (**c**) PCBs set on generator device.

After the circuits were designed two PCBs were made, one for the power supply (1) and another for the acquisition and data communication (2) as seen in Figure 8a,b. They are attached through terminals that also connect them electrically. This configuration makes the system more versatile, if it is necessary to change the acquisition and communication circuit for another one with more sensors or that has some modifications, just replace the corresponding plate. Figure 8c shows the assembled system with all its parts ready.

#### 2.3.4. Assembly

**Figure 9:**
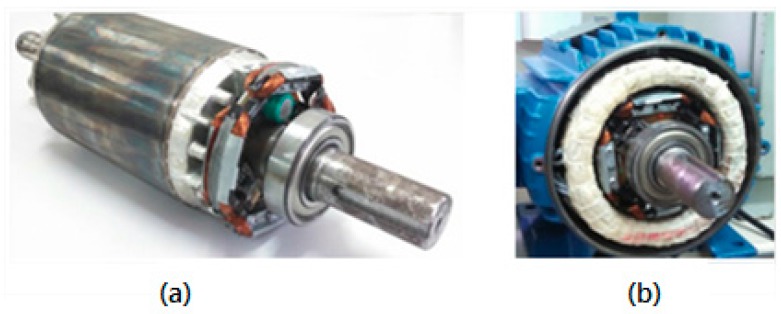
System coupled to the rotor. (**a**) Outside the stator; (**b**) inside the stator.

With the generator built and the circuits designed, the PCBs of the rectifier circuit and the data acquisition and communication circuit were made. The parts were joined and the whole system was coupled to the rotor, as shown in Figure 9. With the machine running the system will rotate along with the rotor and the PCBs will be supplied by energy captured by the generating device.

## 3. Results

The first tests have been done without the presence of the rotor, in order to make a previous analysis of the system behavior. Without the rotor the motor cannot receive the nominal voltage at its terminals, since the inductance is much smaller, consequently it presents a low impedance which would lead to high current values.

To circumvent the problem a VARIAC (Autotransformer) was used, with it was applied a voltage value necessary to generate the nominal current of the motor.

**Figure 10:**
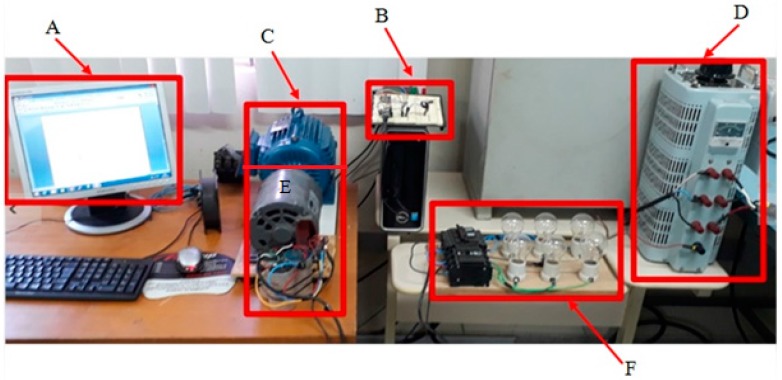
Experimental Bench. (**A**) Computer Monitor; (**B**) Receiver Circuit; (**C**) Motor.; (**D**) VARIAC; (**E**) Generator; (**F**) Load (Lamps).

The tests performed in the laboratory used the structure shown in Figure 10. A is the computer screen that provides the graphics with the temperature values received by the circuit B (connected to the USB port of the computer), sent by the embedded system located inside the motor C which is energized by the three-phase VARIAC D.

In E there is also a gray induction machine, used as a generator that feeds a set of lamps with 180 W of power in F, serving as a load for the motor during laboratory tests.

### 3.1. Generator Rating

The generator device underwent a test to verify if it meets the required voltage and output power (to supply the embedded system). For that a load of 35 Ω corresponding to the internal resistance of the generator was applied, according to the maximum power transfer theorem, it is in this condition that the generator will provide its maximum power.

**Figure 11:**
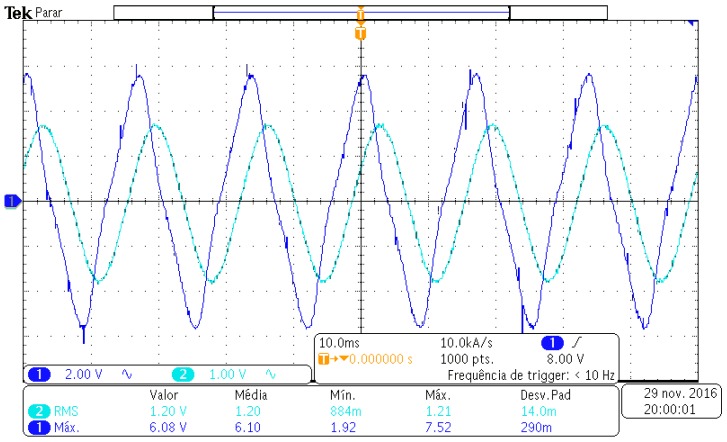
Waveform showing the voltage generated in the generator device.

During the test, with a 12 A_rms_ current applied on the stator winding (light blue waveform), a peak voltage of 6 V (4.24 V_rms_) was measured at the output (dark blue waveform), which corresponds to a power of approximately 500 mW. The system requires at least 5.5 V peak and a minimum power of 165 mW, the result is shown in Figure 11. Therefore the generating device meets the requirements of the system.

**Figure 12:**
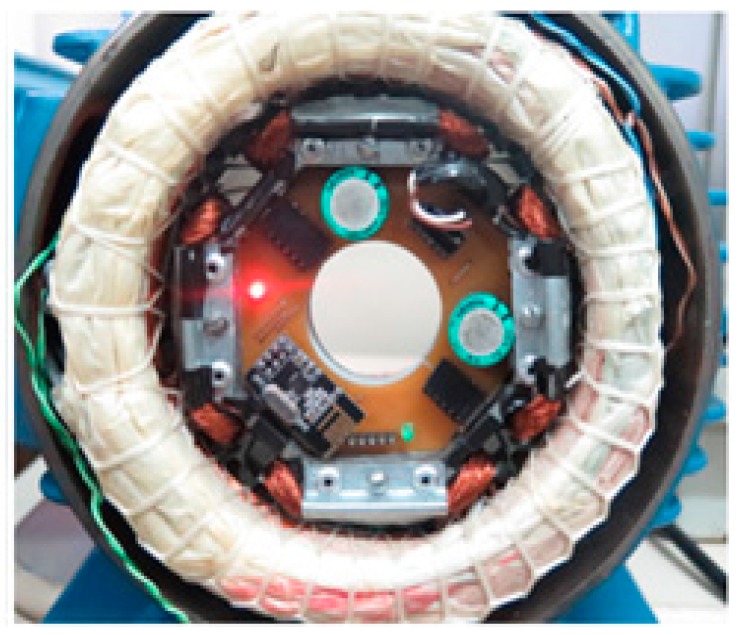
Voltage value generated after passing through the regulator.

The next test was based on the embedded system (PCBs + Prototype) using PCBs as load, which means that all components are powered by the generator device (Figure 12). The voltage at the regulator output reached 3.3 Vdc. In this test, all components: temperature sensor; microcontroller; and RF module are working as proposed.

### 3.2. Evaluation of Data Transmission

**Figure 13:**
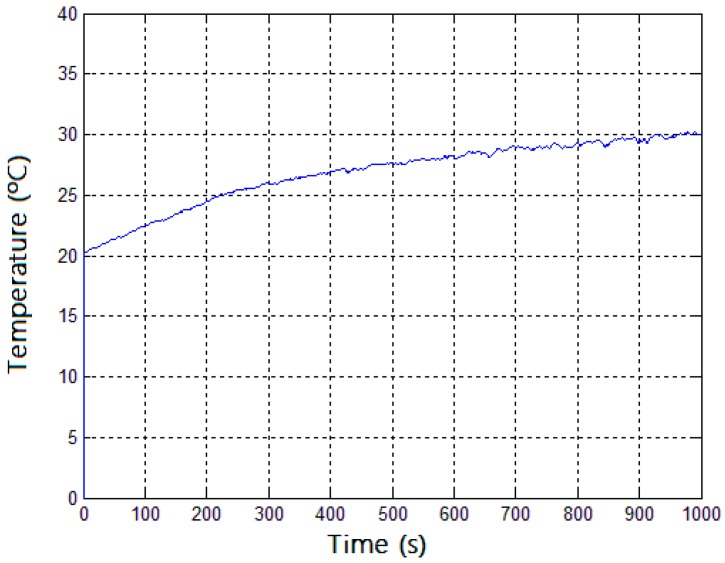
Temperature values, inside the motor, received and plotted on the computer.

In order to verify interference of the magnetic field of the machine on the data acquisition and communication circuit, the embedded system was confined inside the motor after the rear and front covers were placed for interference analysis. The temperature reading, with the transducer in contact with the generator core, is sent to the computer with a sampling rate of one second. Figure 13 shows the temperature values read for 1000 s. The graph shows the motor temperature increases.

The next tests were performed with the system coupled to the rotor and the machine set to operate.

**Figure 14:**
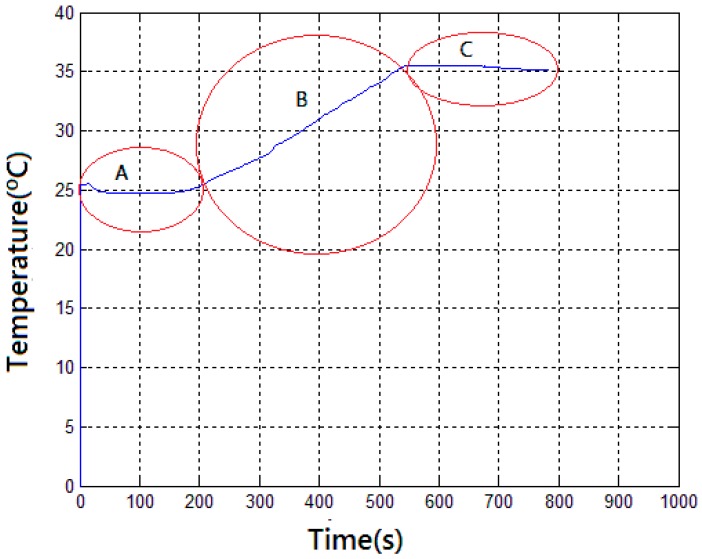
Wireless communication test. A: 50 V in the stator windings; B: 220 V in the stator windings; C: 100 V in the stator windings.

To verify if the rotating radio would interfere with the communication, a test was performed energizing the embedded system with a battery of 12 V and 23 mAh. This battery was chosen because it has dimensions that fit the available space. In order to reduce the power consumption for longer battery life, since it has a low electric charge, this test was done using a time of 2 s between the transmissions, resulting in 793 samples (that corresponds approximately to 26 min) until the complete discharge of the battery as can be seen in Figure 14.

**Figure 15:**
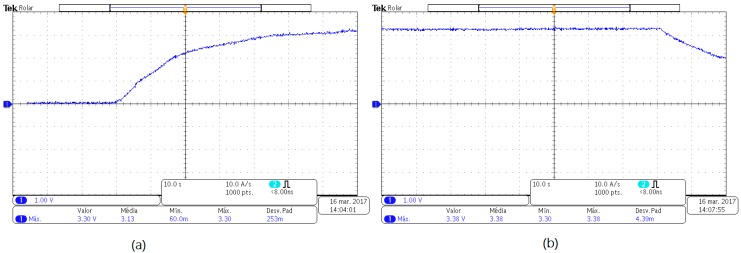
(**a**) Stabilized voltage; (**b**) Voltage after stopping transmitting.

In order to analyze the response of the generating device, a test was performed with the rotor blocked, the stator received a current of 10 A. With the oscilloscope the images for Figure 15 were obtained.

Approximately 25 s after the engine was energized, the computer started to receive the temperature information sent by the prototype. At that time, the voltage in the data acquisition and communication circuit reached 2.42 V. In approximately 70 s the voltage stabilized at 3.3 V until the motor shutdown, as seen in Figure 15b.

**Figure 16:**
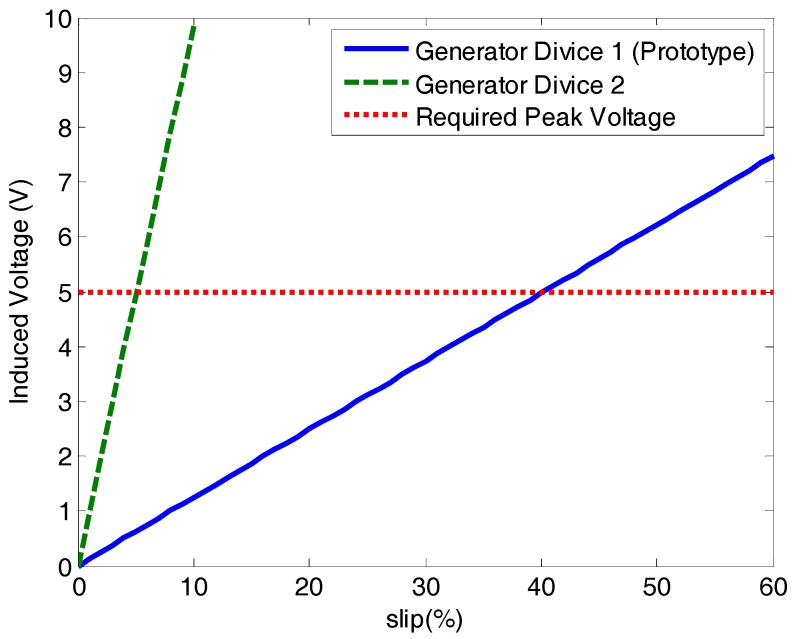
Estimation for the induced voltage in two generating devices with different dimensions.

The generator device has not yet run at rated motor speed, since at this speed the slip frequency is low, about 5% of the synchronous speed. The low slip frequency can be compensated by the increase of the other parameters in Equation (9). Based on Equation (9) and experimental data, an analytical estimate was made for the induced voltage in the generating device for two different situations, the result is in Figure 16.

The straight blue line (Generator Device 1) is the prototype response of this research. This prototype was built based on the dimensions of a 5 HP induction motor as described in Section 2.3.2. It can be noted that it will only produce the voltage necessary for the load at a slip frequency of approximately 40%. The Generator Device 2 would be the response of a generator with at least double the dimensions, 220 turns and installed in a machine of at least 10 HP. This device can already feed the load at a slip frequency of 5% satisfying the rated operating conditions of an induction machine.

In the next step of the research, the data acquisition and transmission circuit will be redone so that it can transmit not only the temperature but also the induced voltage in the generating device. This can be easily achieved using one of the analog inputs of the microcontroller along with the use of batteries to power the circuit. This way it will be possible to relate the induced voltage with the parameters of Equation (9) including slippage. This study will enable more precise changes in the attempt to make the generating device operate at rated engine speed.

## 4. Conclusions

The research was divided into two parts. The first concerns the development of a data acquisition and communication circuit, which allows the reading of rotor information (in this case the temperature) by rotating inside the motor and transmitting them to the external device, in this case a computer. This circuit worked as expected, several tests proved the operation, as it is verified in the presented results. The second part deals with developing a generator device with the aim of replacing the battery and thus guarantee more autonomy and less cost (purchase of batteries). The generator device has not yet operated under the nominal operating conditions of the machine, it still requires a high slip to operate. Improvements are being studied to achieve operation at the rated speed of the machine. The test with the battery in addition to allowing the validation of the circuit data acquisition and communication was also intended to verify how long the system works being exclusively powered by the battery. Our tests found that an alkaline battery of 12 V and 23 mAh kept the transmission for a period of approximately 26 min. It is a short time for most applications, this justifies the search for alternative ways of feeding this type of system.

The prototype was developed for a small motor (5 HP) with little space available for a larger generator device than the one designed, which directly affects the voltage induced in it, as shown in Equation (9). For a greater asynchronous machine than the one used in this work, the better the viability of the proposed system, because the generating device will have larger dimensions, increasing its radius *r* and its thickness *l*. Since the nominal motor currents will be larger, the flux density *Bo* will also increase. These considerations and other improvements, such as using a more efficient AC/DC circuit, compensate for the decrease in induced voltage caused by the low slip frequency.

## Figures and Tables

**Table 1:** Maximum expected current for the PCB of acquisition and communication of data.

Component	Maximum Current
NRF24L01 (RF Module)	14 mA
PIC18F14K50 (Microcontroller)	12 mA
DS18b20 (Transducer)	4 mA
LED	3 mA
**TOTAL**	**33 mA**

**Table 2:** Motor Parameters.

Nominal Parameter	Value
Power	5.0 HP
Voltage(∆/Y)	220/380 V
Current (∆/Y)	14.1/8.19 A
Speed	1725 RPM
Frequency	60 Hz
